# Quality of life and quality-adjusted life years after stroke in Sierra Leone

**DOI:** 10.1177/17474930241249589

**Published:** 2024-05-07

**Authors:** Daniel Youkee, Gibrilla F Deen, Catherine Sackley, Durodami R Lisk, Iain Marshall, Marina Soley-Bori

**Affiliations:** 1 School of Life Course & Population Sciences, King’s College London, London, UK; 2 College of Medicine and Allied Health Sciences, The University of Sierra Leone, Freetown, Sierra Leone; 3 School of Health Sciences, University of Nottingham, Nottingham, UK

**Keywords:** Stroke, Sierra Leone, Africa, quality of life, quality-adjusted life years, health-related quality of life

## Abstract

**Background:**

Stroke is a leading cause of mortality and negatively affects health-related quality of life (HRQoL). HRQoL after stroke is understudied in Africa and there are no reports of quality-adjusted life years after stroke (QALYs) in African countries. We determined the impact of stroke on HRQoL after stroke in Sierra Leone. We calculated QALYs at 1 year post-stroke and determined sociodemographic and clinical variables associated with HRQoL and QALYs in this population.

**Methods:**

A prospective stroke register was established at the two-principal adult tertiary government hospitals in Freetown, Sierra Leone. Participants were followed up at 7, 90 days, and 1 year post-stroke to capture all-cause mortality and EQ-5D-3L data. QALYs were calculated at the patient level using EQ-5D-3L utility values and survival data from the register, following the area under the curve method. Utilities were based on the UK and Zimbabwe (as a sensitivity analysis) EQ-5D value sets, as there is no Sierra Leonean or West African value set. Explanatory models were developed based on previous literature to assess variables associated with HRQoL and QALYs at 1 year after stroke. To address missing values, Multiple Imputation by Chained Equations (MICE), with linear and logistic regression models for continuous and binary variables, respectively, were used.

**Results:**

EQ-5D-3L data were available for 373/460 (81.1%), 360/367 (98.1%), and 299/308 (97.1%) participants at 7, 90 days, and 1 year after stroke. For stroke survivors, median EQ-5D-3L utility increased from 0.20 (95% CI: −0.16 to 0.59) at 7 days post-stroke to 0.76 (0.47 to 1.0) at 90 days and remained stable at 1 year 0.76 (0.49 to 1.0). Mean QALYs at 1 year after stroke were 0.28 (SD: 0.35) and closely associated with stroke severity. Older age, lower educational attainment, patients with subarachnoid hemorrhage and undetermined stroke types all had lower QALYs and lower HRQoL, while being the primary breadwinner was associated with higher HRQoL. Sensitivity analysis with the Zimbabwe value set did not significantly change regression results but did influence the absolute values with Zimbabwe utility values being higher, with fewer utility values less than 0.

**Conclusion:**

We generated QALYs after stroke for the first time in an African country. QALYs were significantly lower than studies from outside Africa, partially explained by the high mortality rate in our cohort. Further research is needed to develop appropriate value sets for West African countries and to examine QALYs lost due to stroke over longer time periods.

**Data availability:**

The Stroke in Sierra Leone anonymized dataset is available on request to researchers, see data access section.

## Introduction

Stroke is the second leading cause of adult death worldwide and results in significant functional and neurological decline. In Africa, the available epidemiological studies suggest that stroke incidence is increasing and country-level studies demonstrate that stroke survivors have lower health-related quality of life (HRQoL) than matched controls.^1,2^ In Sierra Leone, stroke is associated with high mortality of 49% at 1 year and significant functional impairment;^3^ however, the impact on quality of life is unknown. Sierra Leone is a postcolonial, low-income country in West Africa. Gross Domestic Product (GDP) per capita in 2021 was US$480, in comparison to Ghana US$2363 and neighboring Guinea US$1189.^4^ Sierra Leone ranks 181st out of 195 countries in the UN multidimensional human development index.^5^ Adult life expectancy at birth is 60 years.^6^

A systematic literature review of HRQoL in Africa in 2020 found 28 studies from eight countries, all of low methodological quality with only 14% using a translated-adapted instrument and 11% using an instrument psychometrically validated in the study population.^7^ Pooled global estimates, which do not include any African studies, demonstrate that stroke negatively affects the quality of life of stroke survivors, with stroke survivors reporting lower quality of life than the normal population.^8^ Outside of Africa, HRQoL after stroke is lower in women, older individuals and in the acute phase post-stroke compared to later stage. HRQoL significantly increases from the acute phase to 90 days post-stroke and then stabilizes.^8^ African studies demonstrate similar associations with older age,^9,10^ female sex,^11^ lower educational attainment,^12,13^ and increased stroke severity^9,13^ being associated with lower HRQoL.

The EQ-5D-3L is the most frequently used HRQoL instrument in stroke trials.^14^ It is a simple, generic instrument that evaluates HRQoL across five dimensions, including mobility, self-care, usual activities, pain and discomfort, and anxiety and depression. The EQ-5D-3L was translated into Krio for Sierra Leone, the national lingua franca. Our previous work has shown the Krio EQ-5D-3L to be feasible, with a high completion rate of 81%, 98%, and 97% at 7, 90 days, and 1 year, respectively. The instrument has strong known-group validity by stroke severity^15^ and is responsive to a minimal clinically important difference, representing the smallest improvement deemed worthwhile by the patient,^16^ in the Barthel Index.^17^

As described, stroke is a cause of both mortality and decreased HRQoL. HRQoL and survival data can be combined to estimate quality-adjusted life years (QALYs) which simultaneously measures changes in HRQoL and mortality. To operationalize the QALY concept, the EQ-5D-3L health states can be converted into utility values, using a value set that represents the valuation or preferences for each health state. Dead is anchored at 0 and 1 is perfect health, and a value lower than 0 signifies a state considered worse than death. QALYs have not been reported for stroke populations in Africa before.

This study aims to describe HRQoL after stroke at 7, 90 days, and 1 year. We then calculate QALYs at 1 year after stroke for the first time in an African population. Finally, we present variables associated with HRQoL and QALYs at 1 year post-stroke.

## Methods

### Cohort

A prospective stroke register was established at the two-principal adult tertiary government hospitals in Freetown, Sierra Leone at Connaught Teaching Hospital from 1 May 2019 to 30 September 2021 and at 34th Military Hospital from 1 February 2021 to 2 September 2021. All consecutive patients aged 18 years and above meeting the World Health Organization (WHO) International Classification of Diseases, 10th Revision (ICD-10) definition of stroke^18^ were included. The study methods and the healthcare setting have been previously described in depth.^19^ Strokes were confirmed after review of clinical notes and neuroimaging and stroke type categorized by an experienced stroke physician.

Data were collected on admission and at 7, 90 days, and 1 year post-stroke. All-cause mortality was recorded at each timepoint. The EQ-5D-3L was introduced for follow-up on the 24 February 2020 and at 7 days post-stroke on the 22 June 2020. During admission, interviews were conducted at a private space close to the patient’s bedside, follow-up interviews were conducted by telephone^20^ by research assistants who were native Krio speakers and trained in Good Clinical Practice.^21^ Responses were recorded on paper copies of the case report forms, double data entry was conducted, and all data uploaded onto REDCap™.

### Instruments and definitions

The EQ-5D-3L Interviewer Administered v2.1 was translated into Krio for Sierra Leone using an expanded methodology based on the EuroQol Foundation guidelines,^22^ including focus groups, a process of iterative forward and backward translation, two independent forward translations and two independent back translations, followed by review and reconciliation. The translated tool can be accessed here.^23^ If the patient was unable to answer the questionnaire, proxy response was sought from the principal caregiver. Stroke severity was measured by the National Institute of Health Stroke Scale (NIHSS),^24^ by trained clinicians, as a continuous variable and was categorized into mild stroke (NIHSS < 8), moderate stroke (NIHSS 8–15), and severe stroke (NIHSS > 15). Education was categorized into higher and lower educational attainment (completed primary school or higher). Breadwinner referred to the primary income generator of the household. Hypertension was defined as blood pressure ⩾ 140/90 mmHg from 72 h after stroke or; patient-reported history of hypertension or; history of antihypertensive use or continuing use of antihypertensives 72 h post-stroke. Type II diabetes mellitus was identified based on previous documented history of diabetes or; previous reported history of diabetes; previous prescription of diabetic medication; or HbA1C > 6.5% on admission.

There is no EQ-5D value set for Sierra Leone or for any West African country, although recent value sets for the five-level version (EQ-5D-5L) as opposed to the three-level version (EQ-5D-3L) have been created in Ethiopia and Uganda.^25^ We therefore used the UK value set as the base analysis because it was generated using a large sample size,^26^ linguistic links between English and Krio, and the source questionnaire for our translation was in English. When appropriate throughout the article, we display utility values calculated using the Zimbabwe value set and present a full sensitivity analysis using the Zimbabwe value set in the supplementary material. QALYs were calculated at the patient level using EQ-5D-3L utility values and survival data from the register, following the area under the curve method.^27^ We assumed a linear relationship between utilities at different timepoints. No discounting was applied as our time horizon was 1 year. HRQoL is shown graphically, at 7, 90 days, and 1 year post-stroke using violin plots. Violin plots are a hybrid of box plots and density plots that demonstrate both the distribution and density of the data points. The wider the curve, the higher the density of datapoints within that particular range.

### Missing data

The dataset was assessed for the amount and patterns of missing data, including missing completely at random (MCAR), missing at random (MAR), and missing not at random (MNAR).^28^ There is no official guidance from the EuroQol group on missing data replacement strategies; however, recent studies have shown that mixed-effects repeated measure models (MMRM) and Multiple Imputation methods provide similar results.^29^ We conducted Multiple Imputation using chained equations (MICE), imputing 50 datasets, with all the variables from the outcome model included in the imputation model to ensure that the imputation model preserved the relationships between the variables of interest.^30^ We also included variables not in the analysis model but associated with loss to follow-up, such as district of residence. We imputed continuous variables using linear regression models and binary variables using logistic regression models. We did not transform skewed variables before imputation. Imputed data were checked against observed data graphically.

### Regression model development

Explanatory models were developed to assess variables associated with HRQoL and QALYs at 1 year after stroke. Model development was informed by theory, our previous work,^3,31^ and the available global^8^ and African literature.^7^ The justifications for variable selection, model evolution, and diagnostics are reported in the Supplementary Table S10. We selected age and NIHSS as control variables, and investigated the associations of sex, stroke type, first ever stroke versus previous stroke, comorbidities, and socioeconomic factors. Univariable and multivariable regression analyses were conducted, and we report coefficients and 95% confidence intervals.

We conducted two sensitivity analyses: a sensitivity analysis using EQ-5D Zimbabwe value set and a complete case analysis. In addition, we present HRQoL results assigning a utility value of 0 to patients who died, Supplementary Table S9.

### Ethical approval

The study received ethical approval from King’s College London (HR-18/19-8467) and approval from the Sierra Leone Ethical and Scientific Review Committee on 18 December 2018. All participants, or their family members for participants who lacked capacity, provided written consent to the research.

## Results

The case fatality rate was 24.5% at 7 days, 44.4% at 90 days, and 49.9% at 1 year.^3^ EQ-5D-3L was completed in 373/460 (81.1%), 360/367 (98.1%), and 299/308 (97.1%) of eligible patients at 7, 90 days, and 1 year, respectively.

The median EQ-5D-3L utility value in stroke survivors was 0.20 (95% CI: −0.16 to 0.59), 0.76 (0.47 to 1.0), and 0.76 (0.49 to 1.0) at 7, 90 days, and 1 year post-stroke, respectively, (Table 1). EQ-5D-3L health states, response rate, missing item data, and visual analogue scales at 7, 90 days, and 1 year post-stroke are reported in Supplementary Tables S1 and S2. Utilities calculated using the Zimbabwe value set were higher at 7 days than with the UK value set, Supplementary Figure S1 and Table S3.

**Table 1.** EQ-5D-3L utility values after stroke at 7, 90 days, and 1 year in survivors using UK and Zimbabwe value sets.

	UK median (IQR)	Zimbabwe median (IQR)
7 days post-stroke, n = 373	0.20 (−0.16 to 0.59)	0.50 (0.26 to 0.66)
90 days post-stroke, n = 360	0.76 (0.47 to 1.0)	0.77 (0.60 to 1.0)
1 year post-stroke n = 299	0.76 (0.49 to 1.0)	0.77 (0.61 to 1.0)

The EQ-5D-3L utility values for all patients, assigning 0 to those who died, are presented in the Supplementary Table S8 and Figure S3.

**Figure 1.**
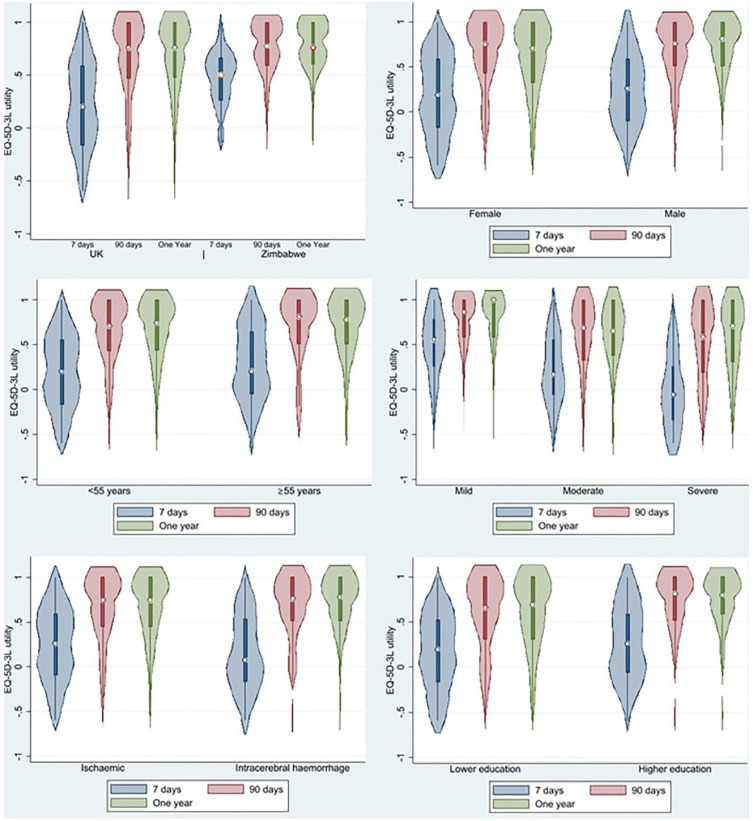
Violin plots of EQ-5D-3L utility at 7 days (blue), 90 days (red), and 1 year (green): (a) UK utility value versus Zimbabwe value set; (b) female versus male sex; (c) age < 55 versus ⩾ 55 years; (d) stroke severity on admission mild stroke (NIHSS < 8), moderate stroke (NIHSS 8–15), and severe stroke (NIHSS > 15), (e) ischemic versus intracerebral hemorrhage, and (f) higher educational attainment versus lower educational attainment.

Male stroke survivors, patients < 55 years, survivors of mild strokes, and ischemic strokes, and those with higher educational attainment reported higher HRQoL (Figure 1).

Median QALYs at 1 year after stroke were 0.07 (IQR: 0.0 to 0.59), for mild stroke 0.62 (0.32 to 0.86), moderate stroke 0.35 (0.01 to 0.67), and severe stroke 0.0 (−0.00 to −0.09) (Table 2). QALYs calculated using the Zimbabwe value set were significantly higher, Supplementary Table S4.

**Table 2.** 1-year QALYs by stroke severity and stroke type using UK value set and MICE dataset, n = 986.

	Median (IQR)	Mean (SD)
All	0.07 (0.0 to 0.59)	0.28 (0.35)
Mild stroke	0.62 (0.32 to 0.86)	0.55 (0.34)
Moderate stroke	0.35 (0.01 to 0.67)	0.35 (0.34)
Severe stroke	00 (−0.00 to −0.09)	0.12 (0.25)
Ischemic	0.27 (0.0 to 0.65)	0.33 (0.36)
Intracerebral hemorrhage	0.06 (0.0 to 0.64)	0.28 (0.36)
Subarachnoid hemorrhage	0.0 (0.0 to 0.49)	0.20 (0.35)
Undetermined stroke type	0.0 (0.0 to 0.0)	0.03 (0.15)
Higer education attainment	0.24 (0.0 to 0.68)	0.34 (0.37)
Lower educational attainment	0.01 (0.0 to 0.51)	0.24 (0.33)
Breadwinner	0.24 (0.0 to 0.68)	0.34 (0.37)
Not breadwinner	0.01 (0.0 to 0.49)	0.23 (0.33)

The results of the multivariable models predicting HRQoL and QALYs at 1 year after stroke of the imputed dataset are shown in Table 3. Subarachnoid hemorrhage and undetermined stroke type are associated with decreased HRQoL, while being the primary breadwinner, hypertension and first-ever stroke were associated with higher HRQoL. Higher educational attainment was associated with increased QALYs, and subarachnoid hemorrhage and undetermined stroke types were associated with decreased QALYs.

**Table 3.** Multivariable regression results predicting HRQoL and QALYs at 1 year post-stroke, n = 986.

Variable	Association with HRQoL	Association with QALYs
Age (each additional year)^a^	**−0.003 (−0.006 to −0.002)**	**−0.003 (−0.005 to −0.002)**
Male sex	−0.0144 (−0.069 to −0.040)	−0.027 (−0.069 to 0.015)
Higher education	0.043 (−0.008 to 0.094)	**0.051 (0.011 to 0.091)**
Breadwinner	**0.082 (−0.026 to 0.137)**	0.042 (−0.001 to 0.085)
Hypertension	**0.079 (0.013 to 0.145)**	0.034 (−0.018 to 0.086)
Diabetes	−0.040 (−0.098 to 0.017)	−0.033 (−0.022 to 0.089)
First-ever stroke	**0.080 (0.011 to 0.149)**	0.033 (−0.022 to 0.089)
NIHSS (each additional point)^a^	**−0.017 (−0.020 to −0.014)**	**−0.017 (−0.019 to −0.015)**
Stroke type (versus ischemic)		
Intracerebral hemorrhage	−0.014 (−0.077 to 0.050)	−0.026 (−0.075 to 0.022)
Subarachnoid hemorrhage	**−0.154 (−0.299 to −0.009)**	**−0.143 (−0.261 to −0.0417)**
Undetermined	**−0.113 (−0.190 to −0.037)**	**−0.102 (−0.163 to −0.042)**

Statistically significant variables are in bold and 95% confidence intervals are presented in parenthesis.

a Control variables in the model.

A sensitivity analysis reporting these results using the Zimbabwe value set is presented in the Supplementary Table S5. The use of value set did not significantly change the results, all variables maintained their significance or non-significance and direction of effect, but did influence the absolute values with Zimbabwe utility values being higher, with fewer utility values below 0. A sensitivity analysis using complete case analysis presented similar findings to those in the main article, Supplementary Tables S6 and S7 and Figure S2. We also present HRQoL results assigning a utility value of 0 to patients who died to produce comparable data to other studies, which predictably generated lower median HRQoL scores, Supplementary Table S8 and Figure S3.

## Discussion

This is the first study to report quality of life after stroke in Sierra Leone. We found a similar time trend in HRQoL after stroke to global reports,^8^ with utility being the lowest at 7 days post-stroke, substantially increasing to 90 days and then remaining stable at 1 year. The average utility increased over time both due to improvements at the individual level and due to death of patients with lower utility scores. The EQ-5D-3L utility in stroke survivors at 90 days was 0.76 (IQR: 0.47 to 1.0), higher than global pooled estimates of the EQ-5D-5L for all stroke types 0.68 (95% CI 0.61 to 0.76) and 0.55 (95% CI: 0.43 to 0.68) for ischemic stroke.^32^ This may be partially explained by the younger age of onset of stroke in our sample, mean 59 years, compared to other global cohorts. However, when assigning 0 for patients who died, the median 90-day utility is 0.0 (0.0 to 0.69), which is much lower than the global pooled estimates of studies also assigning 0 to patients who died 0.50 (95% CI: 0.33 to 0.67).^8^ This reflects the high case fatality rate of 49% at 1 year in our cohort, considerably higher than global estimates^33^ and pooled estimates in Africa of 33.2% (95% CI: 23.6 to 44.5).^34^ The high mortality and low HRQoL reported reflect the severe case mix in our cohort. Our cohort presented with a median NIHSS of 16 (9–24), with 396 (40.2%) suffering a stroke-related complication, the most prevalent being aspiration pneumonia. Patients also presented late compared to other settings, the median time from stroke onset to arrival to the hospital was 25 h (IQR 6–73).^31^ At the time of the study, there was no stroke unit or specialized stroke services at either hospital.

This is the first report of QALYs after stroke in Africa and mean QALYs after stroke were substantially lower than estimates from outside Africa. Mean QALYs at 1 year after stroke were 0.28 (SD: 0.35), much lower than the mean value of 0.70 (SD: 0.27) from a UK stroke registry.^35^ This result is likely driven by the higher stroke severity in our cohort, with a median NIHSS of 16 (IQR: 9–24) compared to the UK median 3 (IQR: 0–7).^35^ The low QALYs reported in our study give further urgency to the need to improve access to primary prevention, improve quality, and access of stroke services, such as stroke units, and support to long-term care and rehabilitation in Sierra Leone.^36^ The challenges of primary prevention for stroke in Sierra Leone are considerable. Existing estimates indicate that 33.2% of the population has a diagnosis of hypertension, of which only 14.7% are undergoing treatment and only 4.6% are controlled.^37^ Further research should evaluate novel interventions to improve coverage and quality of primary prevention in Sierra Leone. As a first step toward addressing unmet care need, in 2022, a stroke unit was established at Connaught Teaching Hospital, yet scaling of stroke unit-based care to provide nationwide coverage is needed. This study also demonstrates the impact of stroke on longer-term HRQoL and should focus attention on the optimal design and coverage of post-stroke rehabilitation and support services in our setting.

In concordance with global^8,35^ and African^9,13^ studies, increasing age and stroke severity were both associated with lower HRQoL. We found no difference in HRQoL between patients with ischemic and intracerebral hemorrhage strokes, while patients with subarachnoid hemorrhage and undetermined stroke types were associated with decreased HRQoL compared to patients with ischemic stroke. We did not find any difference in HRQoL between males and females, contrary to studies within^11,13,38^ and outside of Africa.^39,40^ Higher educational attainment, in line with our results, is associated with higher HRQoL after stroke in previous studies within^11–13^ and outside^35,41^ Africa. The variables associated with QALYs were similar to HRQoL predictors. Age, stroke severity, subarachnoid hemorrhage, undetermined stroke types, and lower educational attainment were significantly associated with decreased QALYs, similar to the findings from an Australian stroke registry.^42^

The research is limited by the lack of an appropriate EQ-5D value set. We attempted to mitigate this by conducting the analysis with both the UK and Zimbabwe value sets, but ultimately, we recognize we are not reporting Sierra Leonean or West African preferences. Zimbabwe utility scores were higher at all timepoints and with less scores distributed below 0 than the UK value set but choice of value set made no significant differences to the results of multivariable regression models. The study is limited by calculating QALYs at 1 year and further research should examine the impact of stroke on QALYs at 5 years with the inclusion of a non-stroke reference population which would enable the calculation of QALYs lost due to stroke. Finally, our study is hospital-based and not population-based; therefore, the high case fatality rate and corresponding low QALYs are partially explainable by individuals with mild stroke not accessing care at the tertiary hospitals where the register was based.

To our knowledge, this is the first report of QALYs after stroke in Africa and the study benefits from a well-translated tool, a large sample size, and a prospective design. Incorporating EQ-5D instruments into other African stroke registers will generate comparable data quantifying the burden of stroke and lay the foundation for future economic evaluations to identify cost-effective interventions to improve stroke care and recovery.

## Supplemental Material

sj-docx-1-wso-10.1177_17474930241249589 – Supplemental material for Quality of life and quality-adjusted life years after stroke in Sierra Leone Supplemental material, sj-docx-1-wso-10.1177_17474930241249589 for Quality of life and quality-adjusted life years after stroke in Sierra Leone by Daniel Youkee, Gibrilla F Deen, Catherine Sackley, Durodami R Lisk, Iain Marshall and Marina Soley-Bori in International Journal of Stroke
