# Supplementary material for: Quality of life and quality-adjusted life years after stroke in Sierra Leone
Source: Int J Stroke. 2024 May 7;19(9):981–8. doi: 10.1177/17474930241249589 (PMC11528921; doi:10.1177/17474930241249589)
Supplement: sj-docx-1-wso-10.1177_17474930241249589 – Supplemental material for Quality of life and quality-adjusted life years after stroke in Sierra Leone [file sj-docx-1-wso-10.1177_17474930241249589.docx]

**Supplementary Material**

**Contents**

Page 2 Supplementary Table 1: *EQ-5D-3L health states, response rate, missing item data and visual analogue scales at seven days, 90 days, one year post stroke.*

*Page 3 Supplementary Table 2 Paretian Classification of Health Change for EQ-5D-3L health states from seven to ninety days and from ninety days to one year.*

Page 5 Sensitivity analysis using the Zimbabwe EQ-5D value set

Page 8 Sensitivity analysis: complete case analysis and assigning EQ-5D-3L utility=0 for patients who die

Page 12 Regression model development and justification

|  | **Count** | **Missing** | **No difficulty** | **Moderate difficulty** | **Severe difficulty** |
| --- | --- | --- | --- | --- | --- |
| ***Seven days post stroke n=378*** | | | | | |
| Mobility | 378 | 0 | 86 (22.8) | 138 (36.5) | 158 (40.7) |
| Self-care | 377 | 1 | 73 (19.4) | 155 (41.4) | 149 (35.2) |
| Activities | 377 | 1 | 44 (11.7) | 127 (33.7) | 206 (54.6) |
| Pain | 375 | 3 | 127 (33.9) | 185 (49.3) | 63 (16.8) |
| Anxiety | 373 | 5 | 174 (46.7) | 151 (40.5) | 48 (12.9) |
| VAS | 377 | 1 | Mean 46.1 (SD:21.2) | | |
| ***90 days post stroke n=362 out of 367 contacted (98.6%)*** | | | | | |
| Mobility | 362 | 0 | 218 (60.2) | 117 (32.3) | 27 (7.5) |
| Self-care | 362 | 0 | 214 (59.1) | 115 (31.8) | 33 (9.1) |
| Activities | 362 | 0 | 167 (46.1) | 107 (29.6) | 88 (24.3) |
| Pain | 362 | 0 | 193 (53.3) | 143 (39.5) | 26 (7.2) |
| Anxiety | 362 | 0 | 252 (69.6) | 87 (24.0) | 23 (6.4) |
| VAS | 360 | 1 | Mean 66.0 (SD:21.7) | | |
| ***One year post stroke n=300 out of 308 contacted (97.4%)*** | | | | | |
| Mobility | 300 | 0 | 166 (55.3) | 115 (38.3) | 19 (6.3) |
| Self-care | 300 | 0 | 169 (56.3) | 109 (36.3) | 22 (7.3) |
| Activities | 300 | 0 | 139 (46.3) | 87 (29.0) | 74 (24.7) |
| Pain | 300 | 0 | 186 (62.0) | 94 (31.3) | 20 (6.7) |
| Anxiety | 299 | 1 | 227 (75.9) | 53 (17.7) | 19 (6.4) |
| VAS | 299 | 1 | Mean 71.0 (SD:22.0) | | |

*Table S1 :* *EQ-5D-3L health states, response rate, missing item data and visual analogue scales at seven days, 90 days, one year post stroke.*

|  |  | Change type | At 90 days | | At one Year | |
| --- | --- | --- | --- | --- | --- | --- |
|  |  | No change | Count | % total | Count | % total |
| Mobility | No change | 1 to 1 | 62 | 24.6 | 73 | 38.8 |
|  |  | 2 to 2 | 39 | 15.5 | 21 | 11.2 |
|  |  | 3 to 3 | 13 | 5.2 | 0 | 0.0 |
|  | Worse | 2 to 1 | 46 | 18.3 | 23 | 12.2 |
|  |  | 3 to 1 | 24 | 9.5 | 1 | 0.5 |
|  |  | 3 to 2 | 32 | 12.7 | 7 | 3.7 |
|  | Better | 1 to 2 | 8 | 3.2 | 33 | 17.6 |
|  |  | 1 to 3 | 0 | 0.0 | 0 | 0.0 |
|  |  | 2 to 3 | 4 | 1.6 | 8 | 4.3 |
|  | Death | 1 to death | 2 | 0.8 | 11 | 5.9 |
|  |  | 2 to death | 8 | 3.2 | 9 | 4.8 |
|  |  | 3 to death | 14 | 5.6 | 2 | 1.1 |
| Selfcare | No change | 1 to 1 | 52 | 20.6 | 75 | 39.9 |
|  |  | 2 to 2 | 37 | 14.7 | 22 | 11.7 |
|  |  | 3 to 3 | 9 | 3.6 | 3 | 1.6 |
|  | Better | 2 to 1 | 59 | 23.4 | 18 | 9.6 |
|  |  | 3 to 1 | 22 | 8.7 | 5 | 2.7 |
|  |  | 3 to 2 | 25 | 9.9 | 7 | 3.7 |
|  | Worse | 1 to 2 | 6 | 2.4 | 27 | 14.4 |
|  |  | 1 to 3 | 1 | 0.4 | 2 | 1.1 |
|  |  | 2 to 3 | 7 | 2.8 | 7 | 3.7 |
|  | Death | 1 to death | 1 | 0.4 | 8 | 4.3 |
|  |  | 2 to death | 7 | 2.8 | 11 | 5.9 |
|  |  | 3 to death | 15 | 6.0 | 3 | 1.6 |
| Activities | No change | 1 to 1 | 27 | 10.7 | 42 | 22.3 |
|  |  | 2 to 2 | 18 | 7.1 | 17 | 9.0 |
|  |  | 3 to 3 | 37 | 14.7 | 11 | 5.9 |
|  | Better | 2 to 1 | 47 | 18.7 | 20 | 10.6 |
|  |  | 3 to 1 | 42 | 16.7 | 18 | 9.6 |
|  |  | 3 to 2 | 33 | 13.1 | 13 | 6.9 |
|  | Worse | 1 to 2 | 3 | 1.2 | 16 | 8.5 |
|  |  | 1 to 3 | 4 | 1.6 | 9 | 4.8 |
|  |  | 2 to 3 | 17 | 6.7 | 20 | 10.6 |
|  | Death | 1 to death | 0 | 0.0 | 7 | 3.7 |
|  |  | 2 to death | 7 | 2.8 | 3 | 1.6 |
|  |  | 3 to death | 16 | 6.3 | 12 | 6.4 |
| Pain | No change | 1 to 1 | 60 | 23.8 | 64 | 34.0 |
|  |  | 2 to 2 | 47 | 18.7 | 25 | 13.3 |
|  |  | 3 to 3 | 3 | 1.2 | 0 | 0.0 |
|  | Better | 2 to 1 | 55 | 21.8 | 36 | 19.1 |
|  |  | 3 to 1 | 10 | 4.0 | 6 | 3.2 |
|  |  | 3 to 2 | 14 | 5.6 | 7 | 3.7 |
|  | Worse | 1 to 2 | 28 | 11.1 | 20 | 10.6 |
|  |  | 1 to 3 | 0 | 0.0 | 2 | 1.1 |
|  |  | 2 to 3 | 10 | 4.0 | 6 | 3.2 |
|  | Death | 1 to death | 3 | 1.2 | 11 | 5.9 |
|  |  | 2 to death | 14 | 5.6 | 8 | 4.3 |
|  |  | 3 to death | 2 | 0.8 | 3 | 1.6 |
| Anxiety | No change | 1 to 1 | 95 | 37.7 | 94 | 50.0 |
|  |  | 2 to 2 | 22 | 8.7 | 10 | 5.3 |
|  |  | 3 to 3 | 3 | 1.2 | 0 | 0.0 |
|  | Better | 2 to 1 | 60 | 23.8 | 28 | 14.9 |
|  |  | 3 to 1 | 11 | 4.4 | 11 | 5.9 |
|  |  | 3 to 2 | 8 | 3.2 | 1 | 0.5 |
|  | Worse | 1 to 2 | 20 | 7.9 | 13 | 6.9 |
|  |  | 1 to 3 | 1 | 0.4 | 4 | 2.1 |
|  |  | 2 to 3 | 7 | 2.8 | 5 | 2.7 |
|  | Death | 1 to death | 7 | 2.8 | 13 | 6.9 |
|  |  | 2 to death | 10 | 4.0 | 6 | 3.2 |
|  |  | 3 to death | 6 | 2.4 | 3 | 1.6 |

*Table S2:* *Paretian Classification of Health Change for EQ-5D-3L health states from seven to ninety days and from ninety days to one year.*

**Sensitivity analysis with Zimbabwe value set**

Results using the Zimbabwe value set rather than the UK value set are presented below.

The distributions of the EQ-5D-3L utility values based on each value set are presented in the histograms below. The Zimbabwe value set (red) has less values below zero compared to the UK value set. The values for the same individual are significantly higher in the Zimbabwe value set compared to the UK value set, see table one.


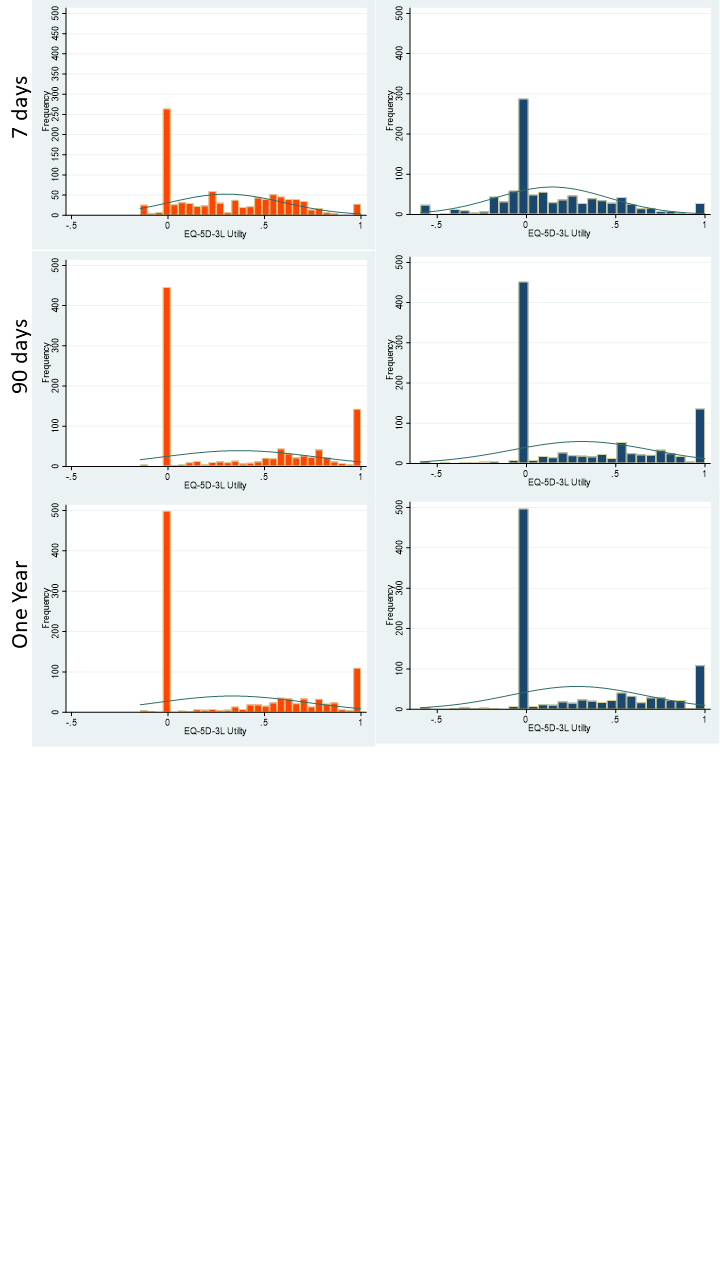


*Figure S1: Histograms of EQ-5D-3L utility values at seven days, 90 days and one year. Zimbabwe value set left and UK value set right. N=986.*

|  | N | UK Median (IQR) | ZW Median (IQR) | P=value | UK Mean (SD) | ZW Mean (SD) | P-value |
| --- | --- | --- | --- | --- | --- | --- | --- |
| Seven days post stroke | 986 | 0.02 (0.00 – 0.35) | 0.24 (0.0-0.55) | <0.001 | 0.14 (0.32) | 0.30 (0.30) | <0.001 |
| 90 days post stroke n=360 | 986 | 0.05 (0.0-0.65) | 0.23 (0.0-0.74) | <0.001 | 0.31 (0.40) | 0.37 (0.40) | <0.001 |
| One year post stroke n=299 | 986 | 0.0 (0.0-0.59) | 0.0 (0.0-0.69) | <0.001 | 0.28 (0.38) | 0.33 (0.39) | <0.001 |

*Table S3: EQ-5D-3L utility values after stroke at seven days, 90 days and one year using UK and Zimbabwe value sets Mean (SD) and paired t test, Median (IQR) and Wilcoxon signed-rank test . N=986*

QALYS are significantly higher overall and for subgroups, using the Zimbabwe value set compared to the UK value set.

*QALYS after stroke are described in table S4.*

|  | *Median (IQR) UK* | *Median (IQR) ZW* | *Significance* | *Mean (SD) UK* | *Mean (SD)* |
| --- | --- | --- | --- | --- | --- |
| *All* | *0.07 (0.0-0.59)* | *0.22 (0.0-0.70)* | *<0.001* | *0.28 (0.35)* | *0.35 (0.36)* |
| *Mild stroke* | *0.62 (0.32-0.86)* | *0.71 (0.50-0.89)* | *<0.001* | *0.55 (0.34)* | *0.63 (0.33)* |
| *Moderate stroke* | *0.35 (0.01-0.67)* | *0.50 (0.01-0.73)* | *<0.001* | *0.35 (0.34)* | *0.45 (0.33)* |
| *Severe stroke* | *0.0 (-0.00-0.09)* | *0.00 (0.00-0.28)* | *<0.001* | *0.12 (0.25)* | *0.17 (0.28)* |
| *Ischaemic* | *0.27 (0.0-0.65)* | *0.47 (0.02 – 0.73)* | *<0.001* | *0.33 (0.36)* | *0.42 (0.36)* |
| *Intracerebral haemorrhage* | *0.06 (0.0-0.64)* | *0.25 (0.0-0.72)* | *<0.001* | *0.28 (0.36)* | *0.35 (0.36)* |
| *Subarachnoid haemorrhage* | *0.0 (0.0-0.49)* | *0.00 (0.00 – 0.55)* | *<0.001* | *0.20 (0.35)* | *0.23 (0.37)* |
| *Undetermined stroke type* | *0.0 (0.0-0.0)* | *0.00 (0.00 – 0.00)* | *<0.001* | *0.03 (0.15)* | *0.04 (0.17)* |
| *Higher education attainment* | *0.24 (0.0-0.68)* | *0.42 (0.01 – 0.75)* | *<0.001* | *0.34 (0.37)* | *0.40 (0.38)* |
| *Lower educational attainment* | *0.01 (0.0-0.51)* | *0.11 (0.00-0.63)* | *<0.001* | *0.24 (0.33)* | *0.31 (0.35)* |
| *Breadwinner* | *0.24 (0.0-0.68)* | *0.44 (0.00 – 0.76)* | *<0.001* | *0.34 (0.37)* | *0.40 (0.38)* |
| *Not breadwinner* | *0.01 (0.0-0.49)* | *0.08 (0.00 - 0.61)* | *<0.001* | *0.23 (0.33)* | *0.30 (0.34)* |

*Table S4: QALYs after stroke comparing UK and Zimbabwe value sets Mean (SD) and paired t test, Median (IQR) and Wilcoxon signed-rank test . N=986*

| Variable | Coefficient (95% CIs) UK Value set | Coefficient 95% Cis ZW value set |
| --- | --- | --- |
| **Age (each additional year)*** | **-0.003 (-0.005 - -0.002)** | **-0.003 (-0.005 - -0.002)** |
| **NIHSS (each additional point)*** | **-0.017 (-0.019 - -0.015)** | **-0.019 (-0.076 – 0.008)** |
| Male Sex | -0.027 (-0.069 – 0.015) | -0.034 (-0.076 – 0.008) |
| **Higher education** | **0.051 (0.011 - 0.091)** | 0.037 (-0.003 – 0.0769) |
| Breadwinner | 0.042 (-0.001 - 0.085) | 0.036 (-0.007 – 0.079) |
| Hypertension | 0.034 (-0.018 – 0.086) | **0.064 (0.012 – 0.012)** |
| Diabetes | -0.033 (-0.022 – 0.089) | -0.042 (-0.088 – 0.003) |
| First ever stroke | 0.033 (-0.022 – 0.089) | 0.053 (-0.003 – 0.108) |
| Stroke type(versus ischaemic) | - |  |
| Intracerebral haemorrhage | -0.026 (-0.075 – 0.022) | -0.040 (-0.088 – 0.009) |
| **Subarachnoid haemorrhage** | **-0.143 (-0.261 - -0.0417)** | **-0.198 (-0.316- -0.080)** |
| **Undetermined** | **-0.102 (-0.163 - -0.042)** | **-0.162 (-0.223 - -0.101)** |
| *Model diagnostics* |  |  |
| *F* | *33.0* | *40.7* |
| *Adjusted R squared* | *0.313* | *0.362* |

*Table S5: Multivariable regression results predicting QALYs at one year after stroke, using Zimbabwe value set n=986. *Control variables*

**Complete case analysis (without assigning 0 to death)**

The EQ-5D-3L utility values of stroke survivors at seven days, 90 days and one year post stroke are presented in *table S6.*

|  | UK Median (IQR) | Zimbabwe Median (IQR) |
| --- | --- | --- |
| Seven days post stroke n=373 | 0.20 (-0.16-0.59) | 0.50 (0.26-0.66) |
| 90 days post stroke n=360 | 0.76 (0.47-1.0) | 0.77 (0.60-1.0) |
| One year post stroke n=299 | 0.76 (0.49-1.0) | 0.77 (0.61-1.0) |

*Table S6: EQ-5D-3L utility values after stroke at seven days, 90 days and one year in survivors using UK and Zimbabwe value sets.*

Violin plots demonstrate HRQoL over time for stroke survivors, and by variable in *figure S2.*


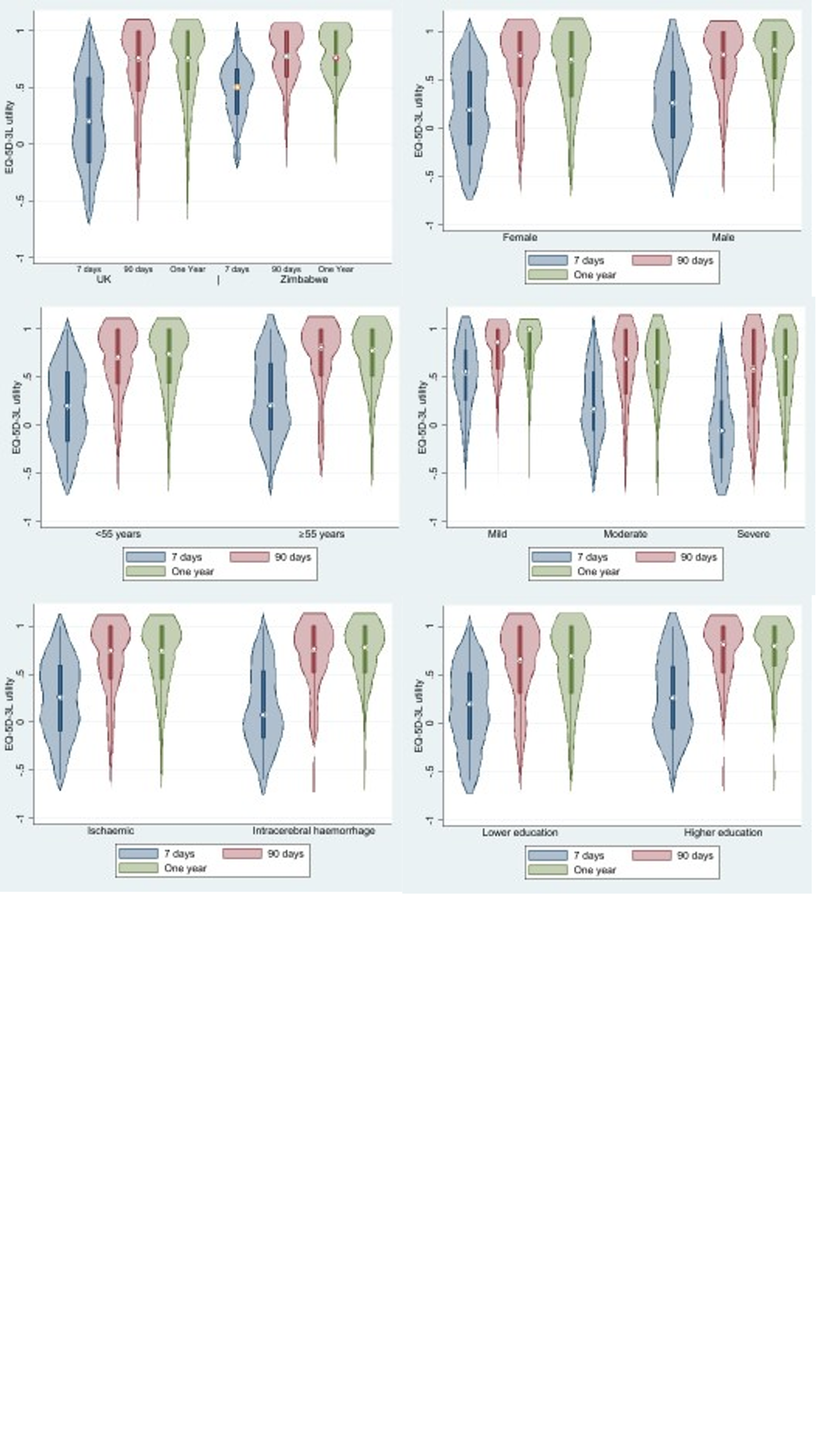


*Figure S2: Violin Plots of EQ-5D-3L utility at seven days (blue), 90 days (red) and one year (green). Figure 1a. UK utility value versus Zimbabwe value set. Figure 1b. Female versus Male sex. Figure 1c. Age <55 years versus Age ≥55 years Figure 1d. Stroke severity on admission mild stroke (NIHSS <8), moderate stroke (NIHSS 8-15) and severe stroke (NIHSS>15). Figure 1e. Ischaemic versus intracerebral haemorrhage. Figure 1f. Higher educational attainment versus lower educational attainment.*

Univariable and multivariable explanatory models of variables associated with EQ-5D-3L utility at one year are presented in *table S7.* Male sex, higher educational attainment and undetermined stroke type were associated with increased HRQoL in the univariable analysis. In the multivariable analysis, similar to the main results, higher educational attainment, being the primary breadwinner and first ever stroke were associated with higher HQRoL whilst stroke severity was associated with lower HRQoL.

| Variable | Univariable | Multivariable |
| --- | --- | --- |
| Age (each additional year)* | **-0.005 (-0.008- -0.001)** | -0.003 (-0.006-0.006) |
| Male Sex | **0.11 (0.02-0.20)** | 0.000 (-0.100-0.100) |
| Higher education | **0.058 (0.015-0.100)** | **0.108 (0.014-0.202)** |
| Breadwinner | 0.013 (-0.019-0.046) | **0.165 (0.963-0.267)** |
| Hypertension | 0.015 (-0.109-0.139) | -0.005 (-0.134-0.125) |
| Diabetes | -0.017 (-0.121-0.088) | -0.014 (-0.125-0.097) |
| First ever stroke | 0.114 (-0.028-0.255) | **0.155 (0.008-0.302)** |
| NIHSS (each additional point)* | **-0.011 (-0.017- -0.005)** | **-0.009 (-0.015—0.003)** |
| Stroke type(versus ischaemic) |  |  |
| Intracerebral haemorrhage | 0.014 (-0.095-0.124) | 0.014 (-0.098-0.125) |
| Subarachnoid haemorrhage | 0.094 (-0.228- 0.415) | 0.106 (-0.202-0.413) |
| Undetermined | **0.646 (0.594-0.698)** | -0.219 (-0.559-0.122) |

*Table S7: Univariable and multivariable explanatory analysis of variables associated with EQ-5D-3L utility at one year post stroke, complete case analysis (n=299), UK value set. *control variables.*

**Complete case analysis, assigning EQ-5D-3L utility=0 for patients who die**

The EQ-5D-3L utility values for all patients at seven days, 90 days and one year post stroke are presented in *table S8.*

|  | N | UK Median (IQR) | Zimbabwe Median (IQR) |
| --- | --- | --- | --- |
| Seven days post stroke | 591 | 0.0 (0.0-0.38) | 0.22 (0.0-0.60) |
| 90 days post stroke n=360 | 798 | 0.0 (0.0-0.69) | 0.0 (0.0-0.75) |
| One year post stroke n=299 | 791 | 0.0 (0.0-0.59) | 0.0 (0.0-0.67) |

*Table S8: EQ-5D-3L utility values after stroke at seven days, 90 days and one year using UK and Zimbabwe value sets*

Violin plots demonstrate HRQoL over time and by variable for all patients with available data in *figure S3.*

*
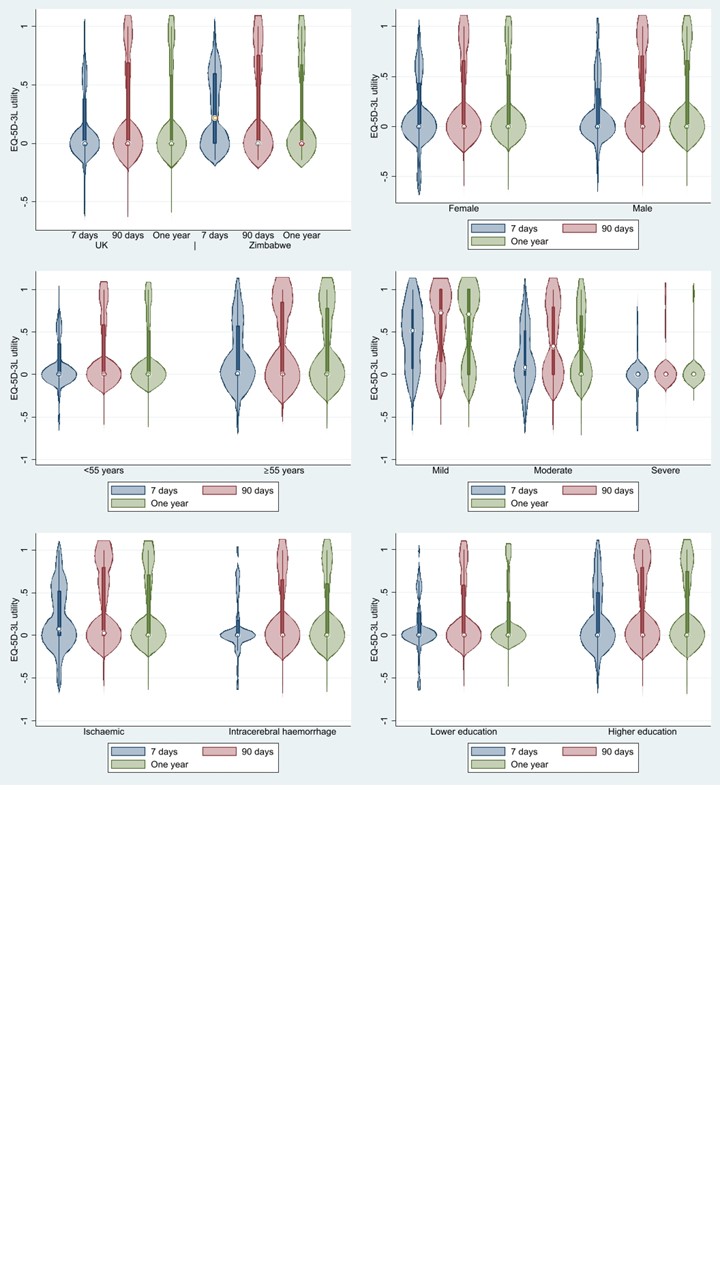
*

*Figure S3: Violin Plots of EQ-5D-3L utility at seven days (blue), 90 days (red) and one year (green). Figure 2a. UK utility value versus Zimbabwe value set. Figure 2b. Female vs Male sex. Figure 2c. Age <55 years versus Age ≥55 years Figure 2d. Stroke severity on admission mild stroke (NIHSS <8), moderate stroke (NIHSS 8-15) and severe stroke (NIHSS>15). Figure 2e. Ischaemic versus intracerebral haemorrhage. Figure 2f. Higher educational attainment versus lower educational attainment.*

Univariable and multivariable explanatory models of variables associated with EQ-5D-3L utility at one year are presented in *table S9.*  Undetermined stroke type were associated with decreased HRQoL and higher education and first ever stroke were associated with increased HRQoL in the univariable analysis. In the multivariable model, undetermined stroke type were associated with decreased HRQoL and being the primary breadwinner, hypertension and first ever stroke were associated with increased HRQoL.

| Variable | Univariable | Multivariable |
| --- | --- | --- |
| Age (each additional year)* | **-0.006 (-0.008- -0.004)** | **-0.004 (-0.006- -0.002)** |
| Male Sex | 0.044 (-0.011-0.010) | -0.024 (-0.079-0.031) |
| Higher education | **0.027 (0.001-0.053)** | 0.052 (-0.001-0.105) |
| Breadwinner | 0.011 (-0.008- 0.031) | **0.083 (0.026-0.140)** |
| Hypertension | 0.058 (-0.014- 0.130) | **0.089 (0.021-0.156)** |
| Diabetes | -0.040 (-0.104-0.02) | -0.042 (-0.101-0.017) |
| First ever stroke | **0.121 (0.044-0.197)** | **0.083 (0.012-0.154)** |
| NIHSS (each additional point)* | **-0.019 (-0.022 - -0.017)** | **-0.016 (-0.019- -0.013)** |
| Stroke type(versus ischaemic) |  |  |
| Intracerebral haemorrhage | -0.031 (-0.100 – 0.038) | -0.002 (-0.069 – 0.064) |
| Subarachnoid haemorrhage | -0.115 (-0.273 – 0.043) | -0.131 (-0.275 – 0.013) |
| Undetermined | **-0.279 (-0.355 - -0.204)** | **-0.091 (-0.167 - -0.014)** |

*Table S9: Univariable and multivariable explanatory analysis of variables associated with EQ-5D-3L utility at one year post stroke, Complete case analysis* assigning EQ-5D-3L utility=0 for patients who die *n=791. * control variables*

**Regression model development**

Model development was informed by theory, our previous work^1, 2^, and the available global^3^ and African literature^4^. A summary of available African literature is presented in *table S10:*

| Variable | Available in SISLE register | Direction of effect on HRQoL based on previous literature |
| --- | --- | --- |
| Age(^5^, ^6^) | Available | Younger>older |
| Gender Male>female (^7-9^) | Available | Male > female |
| Marital status(^10^) | **Unavailable** | Married > unmarried |
| Educational attainment(^11, 12^) | Available | Educated > less educated |
| Employment(^5^) | Available | Employed > unemployed |
| Stroke severity (NIHSS or mRS or stroke levity scale(^13^ ^9^) | Available | Less severe > severe |
| Disability mRS(^10^ ^14^)  Barthel Index(^15^) | Available | Increased function > decreased function |
| Post-stroke depression(^10^,^12^ ^15^) | Available | Not depressed > depressed |
| Co-morbidities   - diabetes^6^ | Available | Diabetic<non-diabetic |

*Table S10: Variables associated with HRQoL in African studies, availability of the variable in the SISLE register and the direction of effect.*

**Model Justification**

There is strong theoretical evidence for stroke severity to be associated with HRQoL, our previous validation the EQ-5D-3L also demonstrated strong known groups validity by stroke severity(NIHSS). There is additionally, good theoretical, global and African literature to support that age will be associated with HRQoL. Our previous work had demonstrated significant associations between age and stroke severity with functional outcome at one year. Therefore we included both age and NIHSS as control variables in our model development.

We experimented with different variable selection and assessed model diagnostics. Due to multicollinearity between Barthel Index prior to stroke and previous stroke, we opted to select previous stroke, as this has more relevance to secondary prevention strategies. Similarly, there was multicollinearity between employment and being the primary breadwinner, we opted for breadwinner to maintain consistency with our previous analyses.

**References**

1. Youkee D, Deen G, Baldeh M, et al. Stroke in Sierra Leone: Case fatality rate and functional outcome after stroke in Freetown. *Int J Stroke* 2023: 17474930231164892. 20230311. DOI: 10.1177/17474930231164892.

2. Youkee D, Deen G, Barrett E, et al. A Prospective Stroke Register in Sierra Leone: Demographics, Stroke Type, Stroke Care and Hospital Outcomes. *Frontiers in Neurology* 2021; 12. Original Research. DOI: 10.3389/fneur.2021.712060.

3. Joundi RA, Adekanye J, Leung AA, et al. Health State Utility Values in People With Stroke: A Systematic Review and Meta-Analysis. *J Am Heart Assoc* 2022; 11: e024296. 20220622. DOI: 10.1161/jaha.121.024296.

4. Bello UM, Chutiyami M, Salihu D, et al. Quality of life of stroke survivors in Africa: a systematic review and meta-analysis. *Qual Life Res* 2021; 30: 1-19. 2020/07/28. DOI: 10.1007/s11136-020-02591-6.

5. Vincent-Onabajo GO, Hamzat TK and Owolabi MO. Consistent determinants of health-related quality of life in the first 12 months after stroke: a prospective study in Nigeria. *Top Stroke Rehabil* 2015; 22: 127-133. 2015/05/06. DOI: 10.1179/1074935714z.0000000033.

6. Donkor ES, Owolabi MO, Bampoh PO, et al. Profile and health-related quality of life of Ghanaian stroke survivors. *Clin Interv Aging* 2014; 9: 1701-1708. 20141008. DOI: 10.2147/cia.S62371.

7. Heikinheimo T and Chimbayo D. Quality of life after first-ever stroke: An interview-based study from Blantyre, Malawi. *Malawi Med J* 2015; 27: 50-54. DOI: 10.4314/mmj.v27i2.4.

8. Akinpelu AO and Gbiri CA. Quality of life of stroke survivors and apparently healthy individuals in southwestern Nigeria. *Physiother Theory Pract* 2009; 25: 14-20. DOI: 10.1080/09593980802622669.

9. Gbiri CA and Akinpelu AO. Quality of life of Nigerian stroke survivors during first 12 months post-stroke. *Hong Kong Physiotherapy Journal* 2012; 30: 18-24.

10. Hamza AM, Al-Sadat N, Loh SY, et al. Predictors of poststroke health-related quality of life in Nigerian stroke survivors: a 1-year follow-up study. *Biomed Res Int* 2014; 2014: 350281. 2014/07/02. DOI: 10.1155/2014/350281.

11. Muli G and Rhoda A. Quality of life amongst young adults with stroke living in Kenya. *Afr Health Sci* 2013; 13: 632-638. DOI: 10.4314/ahs.v13i3.16.

12. Gbiri CA, Akinpelu AO and Odole AC. Prevalence, pattern and impact of depression on quality of life of stroke survivors. *International Journal of Psychiatry in Clinical Practice* 2010; 14: 198-203. DOI: 10.3109/13651501003797633.

13. Vincent-Onabajo G and Adamu A. Impact of poststroke fatigue on health-related quality of life of nigerian stroke survivors. *J Stroke* 2014; 16: 195-201. 20140930. DOI: 10.5853/jos.2014.16.3.195.

14. <Owolabi - 2011 - Impact of stroke on health-related quality of life in diverse cultures The Berlin-Ibadan multicenter international stud.pdf>.

15. Howitt SC, Jones MP, Jusabani A, et al. A cross-sectional study of quality of life in incident stroke survivors in rural northern Tanzania. *J Neurol* 2011; 258: 1422-1430. 20110219. DOI: 10.1007/s00415-011-5948-6.
